# Metabolite and lipoprotein profiles reveal sex-related oxidative stress imbalance in de novo drug-naive Parkinson’s disease patients

**DOI:** 10.1038/s41531-021-00274-8

**Published:** 2022-02-08

**Authors:** Gaia Meoni, Leonardo Tenori, Sebastian Schade, Cristina Licari, Chiara Pirazzini, Maria Giulia Bacalini, Paolo Garagnani, Paola Turano, PROPAG-AGEING Consortium, Alessandra Dal Molin, Anna Bartoletti-Stella, Anna Gabellini, Astrid Daniela Adarmes-Gómez, Cesa Lorella Maria Scaglione, Christine Nardini, Cilea Rosaria, Claudia Boninsegna, Claudia Sala, Cristina Giuliani, Cristina Tejera-Parrado, Daniel Macias, Dolores Buiza-Rueda, Dylan Williams, Elisa Zago, Federica Provini, Francesca Magrinelli, Francesco Mignani, Francesco Ravaioli, Franco Valzania, Friederike Sixel-Döring, Giacomo Mengozzi, Giovanna Calandra-Buonaura, Giovanna Maria Dimitri, Giovanni Fabbri, Henry Houlden, Ismael Huertas, Ivan Doykov, Jenny Hällqvist, Juan Francisco Martín Rodríguez, Juulia Jylhävä, Kailash P. Bhatia, Kevin Mills, Luca Baldelli, Luciano Xumerle, Luisa Sambati, Maddalena Milazzo, Marcella Broli, Maria Giovanna Maturo, Maria Teresa Periñán-Tocino, Mario Carriòn-Claro, Marta Bonilla-Toribio, Massimo Delledonne, Miguel A. Labrador-Espinosa, Nancy L. Pedersen, Pablo Mir, Patrizia De Massis, Pietro Cortelli, Pietro Guaraldi, Pietro Liò, Pilar Gómez-Garre, Robert Clayton, Rocio Escuela-Martin, Rosario Vigo Ortega, Sabina Capellari, Sara Hägg, Sebastian R. Schreglmann, Silvia De Luca, Simeon Spasov, Stefania Alessandra Nassetti, Stefania Macrì, Tiago Azevedo, Wendy Heywood, Claudia Trenkwalder, Claudio Franceschi, Brit Mollenhauer, Claudio Luchinat

**Affiliations:** 1 Magnetic Resonance Center (CERM) and Department of Chemistry “Ugo Schiff”, University of Florence, Sesto Fiorentino, Florence, Italy; 2 Consorzio Interuniversitario Risonanze Magnetiche di Metallo Proteine (C.I.R.M.M.P.), Sesto Fiorentino, Florence, Italy; 3 Department of Clinical Neurophysiology, University Medical Center Goettingen, Goettingen, Germany; 4 IRCCS Istituto delle Scienze Neurologiche di Bologna, Bologna, Italy; 5 Department of Experimental, Diagnostic, and Specialty Medicine (DIMES), University of Bologna, Bologna, Italy; 6 University Medical Center Goettingen, Department of Neurology and Paracelsus-Elena-Klinik, Kassel, Germany; 7 Laboratory of Systems Medicine of Healthy Aging and Department of Applied Mathematics, Lobachevsky University, Nizhny Novgorod, Russia; 8 Personal Genomics Srl, Verona, Italy; 9 Unidad de Trastornos del Movimiento, Servicio de Neurología y Neurofisiología Clínica, Instituto de Biomedicina de Sevilla, Hospital Universitario Virgen del Rocío/CSIC/Universidad de Sevilla, Seville, Spain; 10 Centro de Investigación Biomédica en Red sobre Enfermedades Neurodegenerativas (CIBERNED), Seville, Spain; 11 Consiglio Nazionale delle Ricerche, Roma, Italia; 12 University of Bologna, Bologna, Italy; 13 University of Oxford, Oxford, UK; 14 Karolinska Institutet, Stockholm, Sweden; 15 Department of Biomedical and NeuroMotor Sciences (DiBiNeM), University of Bologna, Bologna, Italy; 16 University of Verona, Verona, Italy; 17 Azienda USL-IRCCS di Reggio Emilia, Reggio Emilia, Italy; 18 Neurologische Klinik, Philipps-University, Marburg, Germany; 19 University of Cambridge, Cambridge, UK; 20 Azienda Unità Sanitaria Locale di Bologna, Bologna, Italy; 21 University College London (UCL), Institute of Neurology, London, UK; 22 UCL Institute of Child Health Library, London, UK; 23 University of L’Aquila, L’Aquila, Italy; 24 S. Maria della Scaletta Hospital, Imola, Italy; 25 Casa di cura Villa Baruzziana, Bologna, Italy

**Keywords:** Predictive markers, Parkinson's disease, Diagnostic markers

## Abstract

Parkinson’s disease (PD) is the neurological disorder showing the greatest rise in prevalence from 1990 to 2016. Despite clinical definition criteria and a tremendous effort to develop objective biomarkers, precise diagnosis of PD is still unavailable at early stage. In recent years, an increasing number of studies have used omic methods to unveil the molecular basis of PD, providing a detailed characterization of potentially pathological alterations in various biological specimens. Metabolomics could provide useful insights to deepen our knowledge of PD aetiopathogenesis, to identify signatures that distinguish groups of patients and uncover responsive biomarkers of PD that may be significant in early detection and in tracking the disease progression and drug treatment efficacy. The present work is the first large metabolomic study based on nuclear magnetic resonance (NMR) with an independent validation cohort aiming at the serum characterization of de novo drug-naive PD patients. Here, NMR is applied to sera from large training and independent validation cohorts of German subjects. Multivariate and univariate approaches are used to infer metabolic differences that characterize the metabolite and the lipoprotein profiles of newly diagnosed de novo drug-naive PD patients also in relation to the biological sex of the subjects in the study, evidencing a more pronounced fingerprint of the pathology in male patients. The presence of a validation cohort allowed us to confirm altered levels of acetone and cholesterol in male PD patients. By comparing the metabolites and lipoproteins levels among de novo drug-naive PD patients, age- and sex-matched healthy controls, and a group of advanced PD patients, we detected several descriptors of stronger oxidative stress.

## Introduction

Parkinson’s disease (PD), after Alzheimer’s disease, is the second most prevalent neurodegenerative disease, affecting 1% of the population over the age of 60^1^, and contributing significantly to the rise in the cost of public health. Despite many years of investigations on metabolic perturbances in PD^2–7^, the mechanisms of aetiopathogenesis, progression, and efficacy of drug treatment on the disease evolution need to be further explored. Currently, routine analytical tests have not yet provided sufficient information to identify reliable blood biomarkers to detect early signs of PD, to monitor the progression of the disease and to detect the effects of therapy intervention.

Metabolomics has become a powerful tool to characterize the biochemistry underlying the onset of different diseases and provides useful applications in the biomedical field.^8–13^. Nuclear Magnetic Resonance (NMR) is one of the most used analytical techniques for metabolomic investigations and it proved to be efficient in characterizing the metabolic composition of different biospecimens in the context of molecular medicine^14–23^. The NMR approach allows one to profile both small metabolites and lipoproteins.

To date, few metabolomic studies focused on de novo drug-naive PD patients (dn^2^PD). Most of them used limited number of subjects (<50)^24,25^ and the results are not always overlapping nor concordant, possibly because of the different analytical approaches or inclusion of PD patients with various phenotypes and stages of PD (genetic or idiopathic PD; de novo or advanced PD patients, etc.)^11,26^, or because of the limited sample size.

**Fig. 1:**
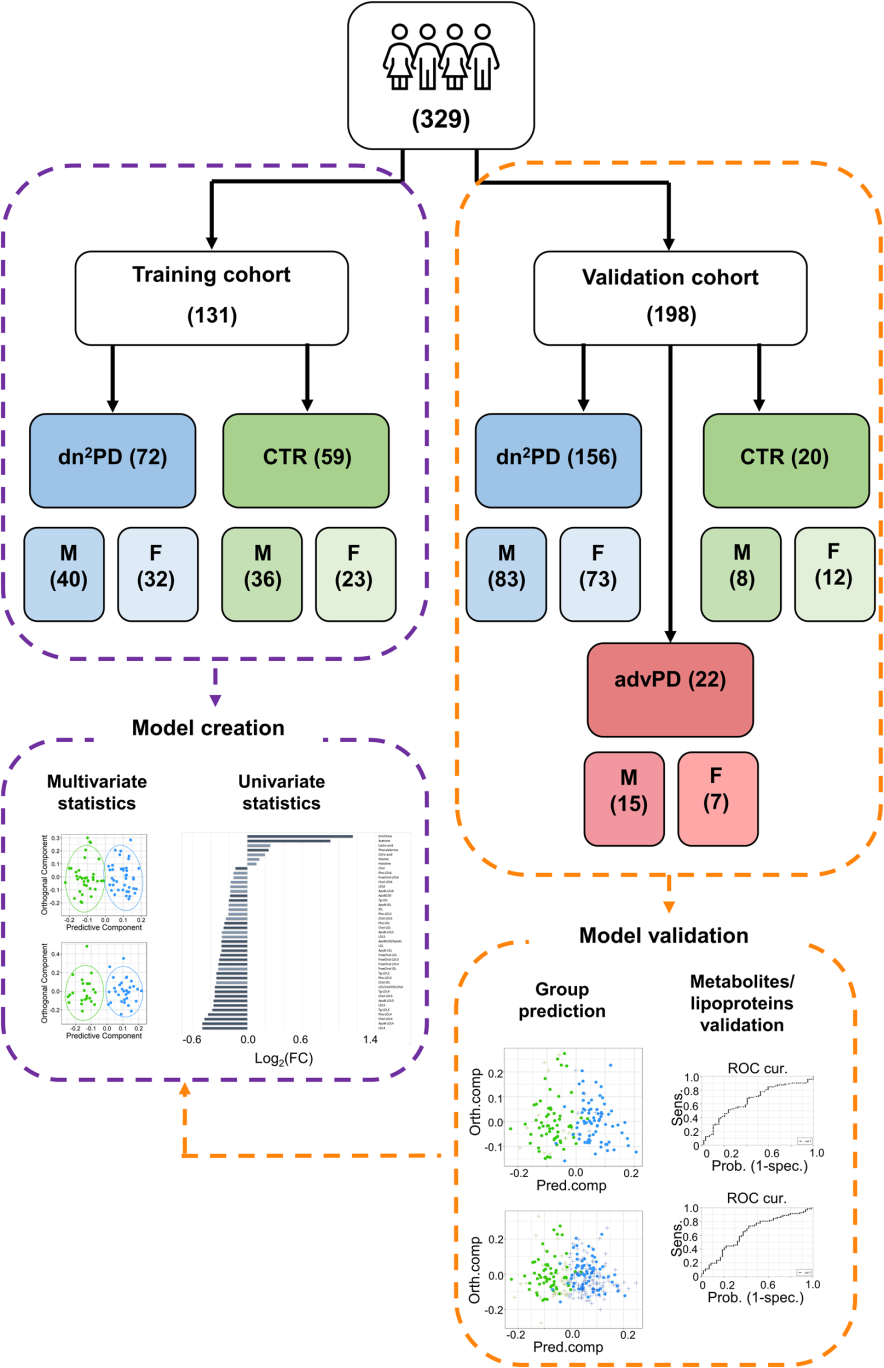
Study design flowchart. The number of subjects for each group (the de novo drug-naive Parkinson’s disease patients (dn^2^PD), healthy control subjects (CTR), and advanced Parkinson’s disease under dopaminergic treatment (advPD)) is reported. The number of male (M) and female (F) subjects for each group are also reported.

In this work, we look for the presence of a metabolomic fingerprint of Parkinson’s disease in the sera of dn^2^PD, and, if present, whether it is sex specific. To do so, we considered 307 subjects, both dn^2^PD (*n* = 228) and healthy controls (CTR, *n* = 79), partitioned between two large independent training and validation cohorts. The peculiar aspect of this collection is the presence of many dn^2^PD patients, to explore the serum biochemistry of the pathology independently of the effects of the antidopaminergic treatment. Additionally, a cohort of 22 advanced PD patients under dopaminergic treatment (advPD) was included to check and test for serum alterations in the disease progression. An overview of the present study design is illustrated in Fig. 1. The subjects in this study are a subset of the cohorts analyzed in the H2020 Project “PROPAG-AGEING” (www.propag-ageing.eu/project)^27^. To our best knowledge, this is the first large NMR-based study with an independent validation cohort aiming at the serum characterization of dn^2^PD patients.

## Results

### Exploratory analysis

**Fig. 2:**
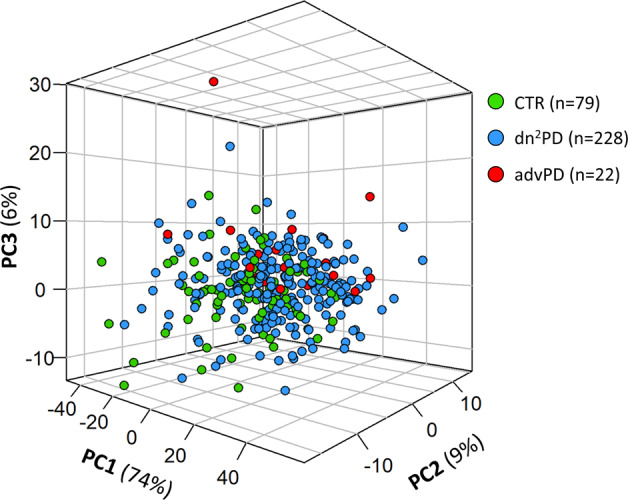
PCA 3D score plot of the whole study population. Each dot represents a 0.02 ppm bucketed 1D-NOESY ^1^H-NMR spectrum color-coded by subject groups.

As a first unsupervised approach, PCA was applied on all available samples (from both training and validation cohorts) to obtain an overview of the variation in the data. Figure 2 shows the PCA 3D score plot on bucketed 1D NOESY spectra color-coded by subject status proving the absence of outlier samples.

### Predictive modeling: OPLS-DA analysis

#### Disease fingerprinting

OPLS-DA analysis was chosen as supervised approach to extrapolate the hidden variables that could be used to characterize the serum fingerprint^28^ (considering the whole NMR spectrum, therefore including the resonance signals arising from metabolites and lipoproteins) of dn^2^PD. First, the training cohort was used to explore differences between dn^2^PDs and CTRs and to derive a discriminant serum fingerprint to correctly assign the new samples according to the diagnosis (dn^2^PD or CTR). The OPLS-DA model built on bucketed serum 1D NOESY spectra of the training cohort showed an evident discrimination between subject groups, featuring an overall mean predictive accuracy of 75.3%, a specificity of 75.0%, and a sensitivity of 75.3% for the classification of dn^2^PD and CTR (Table 1, Supplementary Fig. 1a).

**Table 1:** Performances of the OPLS-DA 1D-NOESY models discriminating dn^2^PD patients from CTR subjects of the training cohort.

	Overall (95% CI) %	Male (95% CI) %	Female (95% CI) %
Accuracy %	75.3 (76.2–74.5)	73.0 (74.2–71.9)	61.6 (63.2–60.1)
Specificity %	75.0 (76.4–74.5)	74.7 (76.2–73.3)	62.1 (63.8–60.4)
Sensitivity %	75.3 (76.3–74.3)	71.3 (72.4–70.2)	61.2 (63.4–58.9)

Overall model (considering all the samples from the training cohort), male and female models (considering separately male and female training groups of cases and controls). Accuracy %, specificity %, and sensitivity % and their confidence intervals (95%) are reported.

#### Sex-dependent fingerprint

Since it has been widely demonstrated that differences between the two sexes could affect manifestation, epidemiology, and pathophysiology of many diseases, and sex discrimination is apparent in metabolomics profiles^29–34^, two independent cross-validated models were created for male (Table 1, Supplementary Fig. 1b) and female training subjects (Table 1, Supplementary Fig. 1c). As shown in Table 1 the male model provides a better discrimination, with an overall predictive accuracy of 73.0% compared to the 61.6% predictive accuracy of the female model.

#### Model validation

An independent cohort was used to validate the existence of a serum metabolic fingerprint differentiating CTRs from dn^2^PD patients. Indeed, the efficacy of the global training model in discriminating dn^2^PD serum samples from CTR was tested using bucketed 1D NOESY spectra from the validation cohort, which were blindly projected onto the OPLS-DA discrimination space obtained from the previously described training model built on both sexes together (Supplementary Fig. 2). New test samples were predicted with an overall accuracy of 74.4%, a specificity of 65%, and a sensitivity of 75.6% (Table 2, Supplementary Fig. 2a, b), thus confirming the presence of a fingerprint discriminating CTRs from dn^2^PD patients. Then, training male and female models were used separately to blindly predict test samples (Table 2). As expected, the model built on the male group performs better in terms of accuracy, sensitivity, and specificity, which are around 70%, while the female model shown completely unbalanced performance values with a sensitivity as low as 41.7% (Table 2).

**Table 2:** Performances of the prediction models.

	Overall (95% CI) %	Male (95% CI) %	Female (95% CI) %
Accuracy %	74.4 (80.7–67.3)	71.4 (80.4–61.0)	75.3 (84.0–64.7)
Specificity %	65 (84.6–40.8)	75.0 (96.8–34.9)	41.7 (72.3–15.2)
Sensitivity %	75.6 (82.2–68.1)	71.1 (80.5–60.1)	80.8 (89.1–69.9)

Table of the averages of the accuracies, the specificities, and the sensitivities of test samples (CTR and dn^2^PD) on the OPLS-DA training models.

##### advPD vs dn^2^PD

Furthermore, OPLS-DA models were created with bucketed 1D NOESY to visualize the discrimination accuracies in the comparison of advPD patients with the dn^2^PD patients and then with CTR subjects. In the first comparison, we obtained an overall mean predictive accuracy of 87.4% (CI = 76.2–95.2%), a mean specificity of 85.1 (CI = 71.4–100%), and a mean sensitivity of 89.7 (CI = 76.2–100%), evidencing a clear discrimination between dn^2^PD and advPD. Optimal performances were obtained also by comparing the advPD patients’ spectra with those from CTRs, resulting in a mean accuracy of 86.9% (CI = 81.6–97.4%), a mean specificity of 87.5% (CI = 78.9–100%), and a mean sensitivity of 86.4% (CI = 78.9–94.7%). Further, projecting onto the OPLS-DA discrimination space built on the training models (dn^2^PD vs CTRs) the 22 bucketed serum 1D-NOESY spectra of advPD subjects, the presence of a serum metabolic signature characterizing healthy from diseased subjects at different stages of the pathology was highlighted. Indeed, all the advPD male and female patients resulted to be correctly classified as PD patients, with an accuracy of 100%. This result supports the idea of the presence of a specific metabolomic signature of PD in sera of affected patients, regardless of the dopaminergic treatment, as the use of PD medication by advPD patients does not influence the classification of those subjects in a model where only patients free from L-DOPA administration are included.

### Metabolite and lipoprotein profiles

#### Profiling of dn^2^PD

**Fig. 3:**
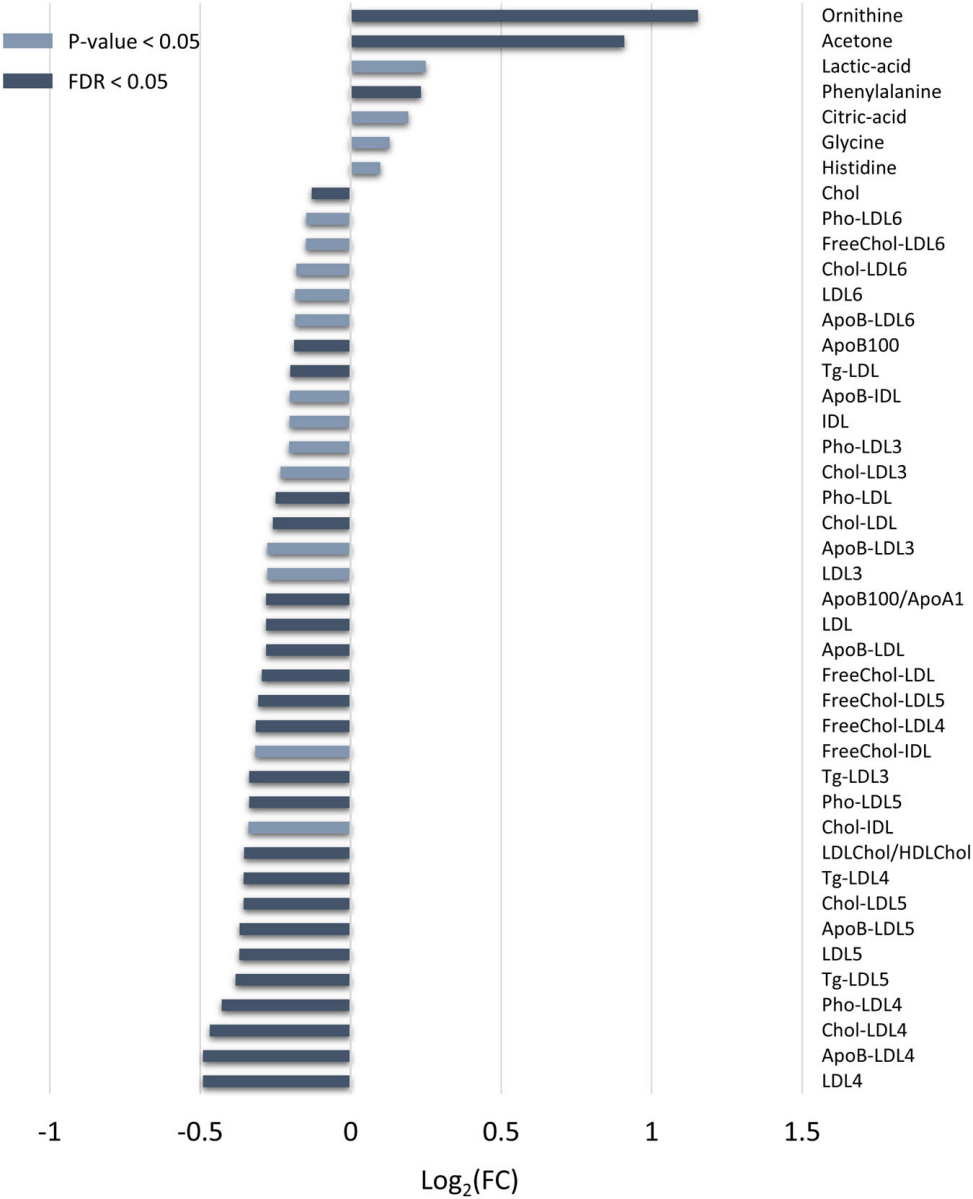
Bar-plot of Log_2_ fold changes values (Log_2_FC). Statistically significant variables (FDR < 0.05) quantified in serum spectra of the male subjects belonging to the training cohort are reported. Negative Log_2_FC values mean higher concentrations in CTR subjects, while positive Log_2_FC values refer to higher concentration levels in dn^2^PD.

Based on multivariate results on NMR spectra, univariate statistics were applied on training cohort samples by keeping subjects separate according to the sex. Corroborating what previously demonstrated by the multivariate models, only male dn^2^PD patients showed a significantly different profile in terms of metabolites and lipoproteins compared to CTR males (Supplementary Table 1). A total of 26 compounds (metabolites and lipoproteins) resulted to be significantly different (FDR < 0.05) in the comparison of male dn^2^PD patients and CTR males: acetone, ornithine, and phenylalanine appear to be significantly higher in dn^2^PD patients with respect to CTRs, while all the 23 significant lipoproteins decrease in the dn^2^PD group (Fig. 3, Supplementary Table 1). Instead, no significant differences were detected when comparing female dn^2^PD patients with female CTRs of the training group, as reported in Supplementary Table 1. Binomial logistic regression models were built using the significant metabolites obtained by comparing the serum profile of male CTR and male dn^2^PD patients of the training cohort (see Fig. 3). The corresponding AUCs (area under the curve) were calculated (Table 3).

**Table 3:** Area under the ROC curve (AUC) values for male subjects of the training and the test set.

	OR (95% CI)	*P* value	FDR	AUC TRAINING	AUC TEST
Ornithine	10.7 (3.02–37.88)	0.0002	0.0021	0.74	0.50
Phenylalanine	9.28 (2.74–31.49)	0.0004	0.0021	0.78	0.54
Acetone	8.43 (2.24–31.74)	0.0016	0.0022	0.80	0.77
Cholesterol	0.18 (0.059–0.56)	0.0031	0.0036	0.70	0.75
LDLChol	0.16 (0.04–0.42)	0.0008	0.0021	0.75	0.66
ApoB100	0.14 (0.04–0.46)	0.0010	0.0021	0.72	0.64
LDLChol/HDLChol	0.14 (0.04–0.44)	0.0008	0.0021	0.73	0.52
ApoB100/ApoA1	0.15 (0.05–0.48)	0.0012	0.0021	0.73	0.52
LDL	0.12 (0.03–0.41)	0.0007	0.0021	0.75	0.67
LDL4	0.14 (0.04–0.46)	0.0012	0.0021	0.72	0.58
LDL5	0.16 (0.05–0.49)	0.0011	0.0021	0.72	0.50
Tg-LDL	0.22 (0.07–0.65)	0.0062	0.0062	0.68	0.61
FreeChol-LDL	0.16 (0.05–0.51)	0.0020	0.0026	0.73	0.67
Pho-LDL	0.12 (0.04–0.42)	0.0008	0.0021	0.75	0.67
ApoBLDL	0.12 (0.04–0.41)	0.0007	0.0021	0.75	0.67
Tg-LDL3	0.17 (0.05–0.54)	0.0025	0.0031	0.72	0.70
Tg-LDL4	0.21 (0.07–0.61)	0.0041	0.0046	0.69	0.50
Tg-LDL5	0.24 (0.08–0.66)	0.0061	0.0062	0.69	0.51
Chol-LDL4	0.15 (0.05–0.48)	0.0015	0.0022	0.72	0.59
Chol-LDL5	0.15 (0.05–0.46)	0.0009	0.0021	0.73	0.50
FreeChol-LDL4	0.19 (0.06–0.61)	0.0046	0.0050	0.70	0.61
FreeChol-LDL5	0.16 (0.05–0.50)	0.0014	0.0022	0.72	0.55
Pho-LDL4	0.15 (0.05–0.48)	0.0015	0.0022	0.72	0.59
Pho-LDL5	0.15 (0.05–0.46)	0.0009	0.0021	0.73	0.50
ApoB-LDL4	0.14 (0.04–0.46)	0.0012	0.0021	0.72	0.58
ApoB-LDL5	0.16 (0.05–0.49)	0.0011	0.0021	0.72	0.50

For training binomial logistic regression models, odds ratio (OR), 95% confidence interval (CI), *P* value, and related values adjusted with the Benjamini–Hochberg correction (FDR) are also reported.

Looking at the discrimination between the two male groups (dn^2^PD and CTR), all the selected metabolites and lipoproteins have a probability between 68.4 and 80.2% to distinguish case (dn^2^PD) from the control (CTR) group. A model based on the concentrations of 27 metabolites and 111 lipoproteins was built to assess its performance in discriminating male dn^2^PD patients and male CTR subjects. In Supplementary Table 2 the performances obtained in the dn^2^PD vs CTR model (training cohort + validation cohort) are compared: (i) using the whole NMR spectrum; (ii) using the concentrations of the identified metabolites and lipoproteins. The former approach appears to perform slightly better (mean accuracy: 77.2%) than the latter (mean accuracy: 73.05%). The list of variable importance in projection (VIP) scores obtained with the latter approach is reported in Supplementary Fig. 3.

##### Profiling disease progression

**Fig. 4:**
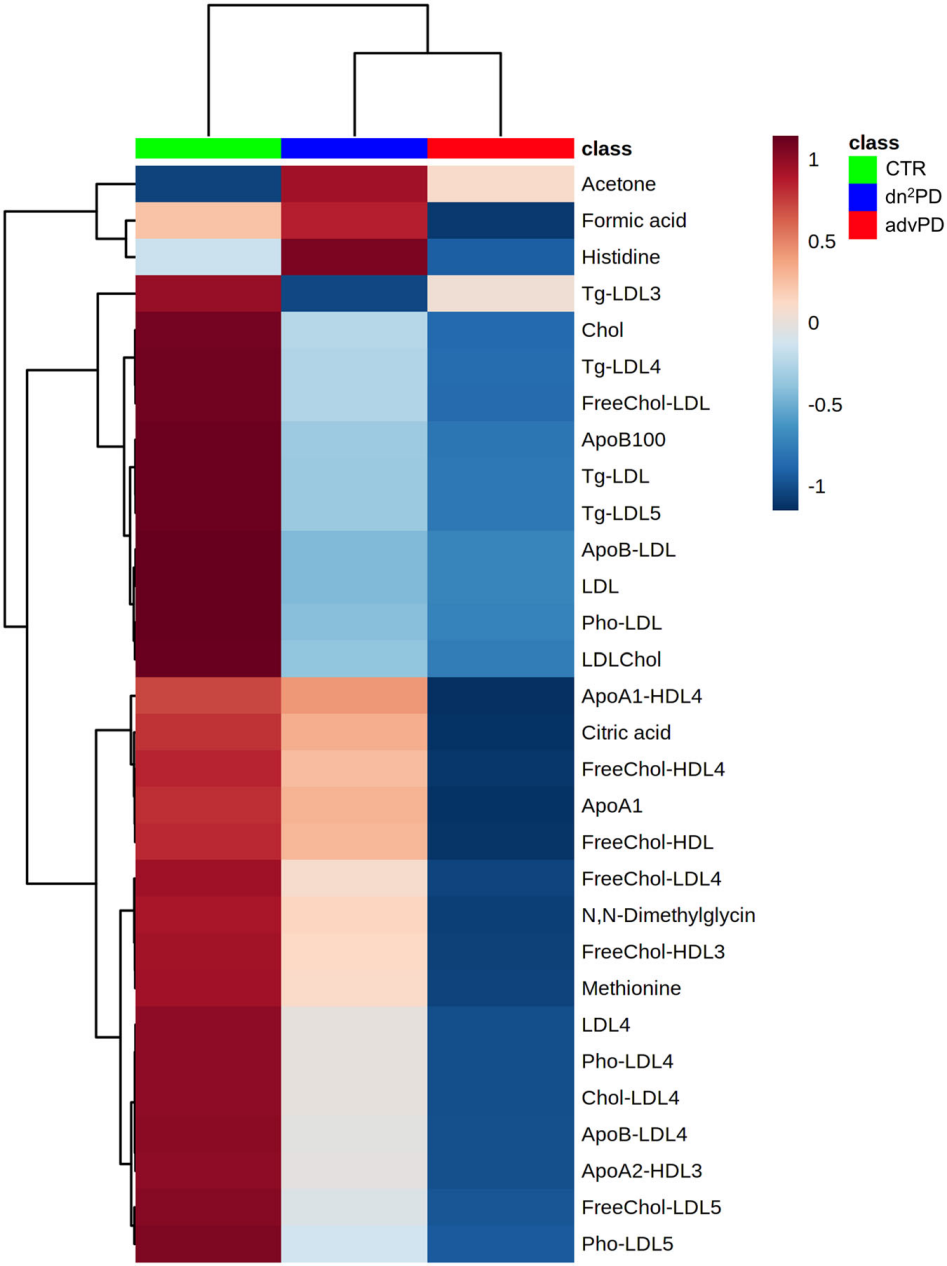
Two-way hierarchical clustering heatmap of the top 30 serum metabolites and lipoproteins. Top features ranked by *t*-test to retain the most contrasting patterns. Heatmap displays average features concentrations for each group (CTR, dn^2^PD, and advPD). The horizontal axis represents the groups, and the vertical axis represents 30 selected features concentrations in which features with similar trends cluster in rows. The magnitude of abundance change (“red” increased or “blue” decreased) is shown in accordance with the color scale on the right.

To explore metabolic variations in more detail, serum metabolic profiles of all dn^2^PD, advPD patients, and CTRs were investigated combining training and validation cohort samples in a single heatmap (both males and females) showing the top 30 analytes identified by *t*-test (Fig. 4). In the two-way hierarchical clustering heatmap, groups and compounds are separated using hierarchical clustering (Ward’s algorithm), with the dendrogram being scaled to represent the distance between each branch (distance measure: Pearson’s correlation). In Fig. 4, a distinct profile signature emerges between PD patients (dn^2^PD and advPD) and CTRs as depicted by the column dendrogram. PD patients are characterized by higher levels of acetone compared to CTRs. Furthermore, the levels of acetone, formic acid, and histidine result to be higher in dn^2^PD than the advPD and CTR subjects. Instead, the PD profile seems to be characterized by lower levels of cholesterol (Chol), low-density lipoproteins (LDL), ApoB100, LDL-cholesterol (esterified and free), phospholipids, and ApoB concentrations in LDL and triglycerides content in LDL fractions (LDL, LDL3, LDL4, and LDL5). Additionally, we notice a marked decrease of ApoA1 lipoprotein and ApoA1 content in the HDL4 fraction, of free cholesterol in the HDL and HDL4 fractions, and of citric acid along the series CTRs>dn^2^PD > advPD. Likewise, a reduction is also observed in the concentration of LDL4, ApoB in LDL4, cholesterol (esterified and free), and phospholipids in LDL4 and LDL5 fractions. Finally, a lowering of ApoA2-HDL3, N,N-dimethylglycine, and methionine also occurs.

## Discussion

To our knowledge, this is the first large NMR-based metabolomics study dealing with the characterization of serum profiles of de novo Parkinson’s disease patients free from dopaminergic treatment compared to healthy sex/age-matched volunteers. In this context, advanced PD patients under dopaminergic treatment are also examined. The use of many well-characterized dn^2^PD and the presence of an external validation cohort represent major strengths of this study. Fingerprinting of the full NMR spectra discriminates dn^2^PD and CTR with a predictive accuracy of 75.3%. The NMR spectra of advPD classify them in the dn^2^PD group with 100% accuracy, proving the existence of a PD serum signature which is clearly recognizable even in the presence of the confounding effect of the dopaminergic therapy.

A more pronounced fingerprint and profile of PD is found in men with respect to women. Increasing evidence points to sex as a crucial determining factor in the development and phenotypical expression of PD. The risk of developing PD is a factor of two higher in men than in women^35,36^. Our observation could support the idea that disease development, at least in dn^2^PD patients, might involve different pathogenetic mechanisms in male and female subjects.

Multivariate analysis detects significant alterations of several metabolites and lipoproteins in PD patients, some of which also survive the scrutiny of univariate analysis. The observation of acetone being higher in dn^2^PD patients reinforces previous studies^8^ and is in line with a general condition of oxidative stress and of increased oxidative stress defense^37,38^. Acetone is a waste product formed from acetoacetic acid. Accumulation of acetone in blood and in breath is common in diabetes, in patients during anesthesia or surgical stress. Other factors such as body weight, age, and especially alcoholism influence the resting blood concentration of acetone^39^. Higher acetone, 3-hydroxybutyrate, and acetate levels have been described also in blood samples from patients with other neurodegenerative diseases, such as multiple sclerosis^40^ and amyotrophic lateral sclerosis^41^. To date, the biological reason for serum acetone to be associated with the onset of PD remains not completely elucidated.

Formic acid and histidine are significantly higher in dn^2^PD when compared with advPD patients and CTRs. Formic acid has been classically identified as mitochondrial toxin and could be also potentially implied in the dopaminergic pathway, explaining the lower level of this molecule in patients under L-DOPA treatment. Instead, literature data report a controversial role of histidine regarding its pro-/anti-oxidant activity^8,42^. Increased concentrations of histidine and phenylalanine have been recently observed also in saliva of PD patients^43^, thus suggesting alterations in neurotransmitters, especially dopamine^44^. Other detected metabolites, such as citric acid, methionine, and N,N-dimethylglycine, playing important roles against oxidative stress damage^45–47^, have been found downregulated in our PD groups. Citric acid alteration could explain the interplay between oxidants and energy metabolism in PD^48^. Methionine and N,N-dimethylglycine are produced by the betaine-homocysteine methyltransferase from homocysteine and betaine. Other studies suggest accumulation of homocysteine in blood and CSF as a risk factor for PD and dementia^49,50^, and this could explain lower levels of the end products in PD patients.

Increased levels of ornithine have been previously detected in serum samples of de novo PD and advanced PD patients^6^. Here, we found higher level of ornithine only in the male dn^2^PD group compared to CTR subjects in the training cohort. However, this metabolite is not significantly altered in the male validation cohort, nor was it found to be altered in the dn^2^PD female population.

Several studies pose lipids and lipoproteins as central players in Parkinson’s disease^51–58^. Various subclasses of fatty acids, glycerolipids, phospholipids, sterol, and lipoproteins may contribute to PD pathogenesis. However, controversial, fragmented and not always reproducible data are available in the literature. Mollenhauer et al.^59^ reported lower serum cholesterol levels in PD patients, independent of nutritional status and body mass index (BMI); dysregulation of cholesterol trafficking was shown to be involved in the pathogenesis of neurodegeneration in PD. Higher cholesterol and LDL-Chol levels might attenuate neurodegenerative process in PD-affected males^60^. Consistently, we found lower cholesterol levels in male patients at early PD stage. For the first time, we also characterize the lipoprotein subclasses profile of serum samples of PD patients. Our results point to a statistically significant decrease of the concentration of small low-density lipoproteins in dn^2^PD patients. Lower LDL-related parameters, especially LDL-cholesterol levels, have been associated with higher PD risk^52,53,61^ and generally, small dense LDL particles (sdLDL) are more susceptible to oxidation than larger LDLs. Therefore, we suggest that sdLDL particles may provide an optimal substrate for ROS oxidative action, which appears to be increased in dn^2^PD patients^54,55,58^.

An interesting result of the present research is that females have a much weaker PD fingerprint than males. Other investigations have found that there are differences in the serum profiles of PD males and females. Because of the scarcity of sex-specific analyses in PD research, the complicated connection between sex and metabolism in relation to PD remains unknown. Further research should consider the modulatory effect of sex hormones, blood metabolites, and PD.

Our data indicate serum alterations which are consistent with increase oxidative stress in PD, suggesting that the early stage of PD may be characterized by increased oxidative defenses and a worsening of the oxidative stress status. The signature of such process is detectable in circulating metabolites and lipoproteins at the very beginning of the clinical onset of the disease. Oxidative stress imbalance bridges the disease with the aging process, the major risk factor for PD. Thus, this result can be somehow interpreted as a peculiar sign of accelerated PD ageing. Of course, further biological investigations are needed to explore in more detail the peripheral signature of ROS stress and PD pathogenesis in Central Nervous System. Our results also support the presence of a complex interaction between sex and metabolism in relation to PD.

## Methods

### Patient cohorts

The study population consists of a total of 329 German subjects, including dn^2^PD patients, advPD patients, and healthy CTR. In detail (Fig. 1), patient cohorts included: (1) a training cohort, consisting of 72 dn^2^PD and 59 CTR, for a total of 131 subjects from the baseline visit of the Kassel cohort, as previously published^27,59,62,63^; (2) an independent validation cohort consisting of samples from 156 dn^2^PD, 20 CTR, and 22 advPD patients, for a total of 198 subjects, as part of the cross-sectional Kassel cohort^27^. Patients enrolled in this study were clinically phenotyped before sample collection. Phenotyping included 1.5 Tesla magnetic resonance imaging (MRI) to determine structural abnormalities, quantitative levodopa testing as published^64^, smell identification test (Sniffin’ sticks, Burghardt Messtechnik GmbH, Wedel, Germany), Mini Mental Status Examination (MMSE) followed by further cognitive testing and video-supported polysomnography to determine REM sleep behavior disorder in a subset of patients. The phenotyping was done based on these results and in accordance to established criteria for PD (UK Brain Bank Criteria)^65^, multiple system atrophy (MSA)^66^, dementia with Lewy bodies (DLB)^67^, progressive supranuclear palsy (PSP)^68^, corticobasal degeneration (CBD)^69^, Alzheimer’s disease and frontotemporal dementia (FTD)^70^. Subjects with marked vascular lesions in MRI indicative of a vascular comorbidity and subjects with normal pressure hydrocephalus by MRI were excluded.

A complete overview of the demographic characteristics of analyzed patients is reported in Table 4. The study was conducted according to the Declaration of Helsinki and with informed written consent provided by all subjects. The study was approved by the ethics committee of the Physician’s Board Hesse, Germany (Approval No. FF89/2008 for DeNoPa) and the University Medical Center Goettingen, Germany (Approval No. 9/7/04 and 36/7/02 for Kassel cohort).

**Table 4:** Demographic characteristics of the population under study.

	Training cohort			Validation cohort					
	dn^2^PD	CTR	*P*	dn^2^PD	CTR	*P*	advPD	*P* (advPD vs dn^2^PD)	*P* (advPD vs CTR)
Age	65.1 ± 9.4	64.5 ± 6.9	0.68	65 ± 11.4	71.7 ± 5.1	6 × 10^–5^	68.9 ± 7.3	0.05	0.16
Sex (male/tot)	40/72	36/59	0.53	83/156	8/20	0.26	15/22	0.19	0.07
BMI	27.7 ± 5	26.7 ± 3.8	0.25	27.1 ± 4.9	26.2 ± 3.2	0.35	25.9 ± 3.7	0.25	0.8
UPDRS III	19 ± 10.2	0.4 ± 0.9	7.4 × 10^–24^	23 ± 12.8	/	/	34.4 ± 15.8	0.003	/
Hoehn and Yahr stage	1.8 ± 0.6	0	1.6 × 10^–35^	2 ± 0.8	/	/	3.1 ± 0.6	6.7 × 10^–8^	/
MMSE	28.4 ± 1.3	28.7 ± 1.2	0.27	28 ± 1.9	/	/	23.3 ± 5.4	0.002	/
Subjects taking Chol-lowering drugs/tot)	7/72	3/59	0.32	32/156	/	/	5/22	0.39	/
Diabetes (cases/tot)	4/72	3/59	0.9	21/156	0/20	/	2/22	0.57	/
Uric acid lowering medications	8/72	3/59	0.21	12/156	2/20	0.72	2/22	0.82	0.92

Mean ± standard deviation and *p*-value (*P*) are reported.

### NMR sample preparation and analysis

All blood samples from training and validation cohorts were collected between 8 AM and 9 AM under fasting condition using Sarstedt tubes for serum collection by venous puncture. Tubes with blood samples were centrifuged at 2500 × *g* for 10 min; serum was aliquoted and frozen within 30 min after collection. The samples were stored at −80 °C, transported in dry ice, until analysis following standard procedures for NMR metabolomic studies^28,71,72^. Samples were then prepared at the Magnetic Resonance Center (CERM/CIRMMP) University of Florence, Italy^73^. The analytical preparation of serum samples and their NMR spectra acquisition followed the protocols detailed elsewhere^28^.

For each serum sample, the one-dimensional (1D) NOESY pulse sequence was applied to acquire ^1^H-NMR spectra, using a Bruker 600 MHz spectrometer, with a proton Larmor frequency of 600.13 MHz and equipped with a 5 mm PATXI ^1^H-^13^C-^15^N and ^2^H decoupling probe. The spectrometer includes a z axis gradient coil, an automatic tuning-matching (ATM), and an automatic and refrigerated sample changer (SampleJet). To stabilize at the level of ±0.1 K the sample temperature, a BTO 2000 thermocouple was employed, and each NMR tube was kept for about 5 min inside the NMR probe head to equilibrate at the acquisition temperature of 310 K.

### Spectral processing

Before applying Fourier transform, raw data were multiplied by an exponential line-broadening function of 0.3 Hz. Transformed spectra were automatically corrected for phase and baseline distortions and calibrated to a reference signal (anomeric glucose proton signal at 5.24 ppm), using Topspin 3.2 software (Bruker BioSpin).

Each 1D serum spectrum, in the range of 0.2–10.0 ppm, was bucketed into 0.02 ppm chemical shift segments using AssureNMR (version 2.2) software (Bruker BioSpin). Regions containing the residual water signal (between 4.68 and 4.84 ppm) were removed.

### Serum and lipoprotein identification and quantification

Twenty-seven metabolites and 111 lipoprotein components (Supplementary Table 1) were identified and quantified from 1D ^1^H-NOESY NMR spectra using the AVANCE Bruker IVDr (Clinical Screening and In Vitro Diagnostics research, Bruker BioSpin)^74^ software. For all serum samples, different lipoproteins (VLDL, LDL, IDL, HDL) and different lipoprotein subclasses, classified according to density and size, for a total of 15 subclasses (VLDL-1 to VLDL-5, LDL-1 to LDL-6, and HDL-1 to HDL-4), were detected. For each main class and subclass, reported data consist of concentrations of lipids (total cholesterol, free cholesterol, phospholipids, and triglycerides) contained in each fraction. Concentrations of apolipoproteins ApoA1 and ApoA2 were estimated for HDL class and each relative subclass, while Apo-B concentrations were calculated for VLDL, IDL classes, and all LDL subclasses.

### Statistical analyses

All data analyses were performed using R (version 3.6.1), an open source software for the statistical management of data^75^.

Multivariate data analysis was conducted on bucketed 1D NMR spectra of all available samples to visualize if spectral features contribute to the separation between groups. Principal component analysis (PCA) was used as a first exploratory approach^76^ to visualize the presence of outliers and to investigate, in an unsupervised manner, the data structure both in the training and in the validation cohorts. PCA analysis was performed on data scaled to unit variance.

Orthogonal projections to latent structures discriminant analysis (OPLS-DA) was employed as supervised technique to check for the presence of a serum metabolomic signature of the disease that distinguishes dn^2^PD patients from CTR^77^. The OPLS-DA approach was applied because it is an improvement of the PLS-DA method. Compared to PLS-DA, the advantage of OPLS-DA is that just one component is employed as a class predictor, while the remaining components reflect variations orthogonal to the first predictive component. A regression model is created in OPLS-DA using multivariate data and a response variable that simply contains class information.

When an unbalanced number of subjects was compared, OPLS-DA models were built by reducing groups to the same size by random sampling, thus including in the model an equal number of subjects from each group. The procedure was repeated 100 times and the results averaged over the 100 models. All discriminant and predictive analyses were performed on bucketed 1D-NOESY spectra without prior normalization. The OPLS-DA models were validated by repeated twofold cross validation (2CV) method. The 2CV scheme (detailed in ref. ^78^), briefly consists of two nested loops CV1 and CV2, where CV1 optimize the number of components to be used in the OPLS-DA model, while the CV2 is to assess the final model performance. In the outer loop (CV2) the complete dataset is split into a test set and a training set: the test set is set aside, and the training set is used in the loop CV1. Within CV1 the training set is split into an internal validation set and another training set (this operation is repeated 50 times). This last training set is used to develop a series of OPLS-DA models with different number of latent variables from which the samples in the internal validation set are predicted. In the CV2 the 90% of the data are randomly chosen at each iteration as a training set to build the model. Then, the remaining 10% are tested. The full procedure is repeated 100 times to derive average discrimination accuracy, sensitivity, and specificity and their confidence intervals (95% CI). Overall, for different classifications, accuracy, sensitivity, and specificity were calculated according to standard definitions, by means of a Monte-Carlo (MC) 2CV scheme (R script written in-house).

Moreover, to externally test the efficacy of the training model in discriminating dn^2^PD from CTR subjects, bucketed 1D NOESY spectra of the validation cohort serum samples (dn^2^PD, CTR, and advPD subjects of the validation cohort) were blindly projected onto the OPLS-DA score plot resulting from the training model.

Univariate Wilcoxon test^79^ was employed to compare metabolite and lipoprotein concentrations between patient groups. The Benjamini & Hochberg method^80^ was applied to correct for multiple testing, and adjusted *P*-values (FDR) < 0.05 were considered statistically significant. Log_2_ fold change (FC) ratios of the median intensities were also calculated for all analyses performed. Effect sizes were estimated for group comparisons using Cliff’s delta formulation^81^ (Cd), which contributes to the characterization of the meaningful signals by giving an estimation of the magnitude of the separation in the different comparisons. Magnitude is evaluated using the thresholds provided in Romano et al.^82^, where Cd values < 0.147 are “negligible”, (Cd) < 0.33 are “small”, (Cd) < 0.474 are “medium”, and (Cd) > 0.474 are “large”.

Association between each statistically significant analyte of the training cohort and the disease was assessed and validated using logistic regression in combination with ROC curve analysis. Before performing any analysis, continuous values of the analytes were standardized by centering and dividing by two standard deviations^83^ using the “rescale” function of the R package “arm”.

First, a binomial logistic regression model was built, for each statistically significant analyte found in the training cohort (i.e., a metabolite or a lipoprotein concentration), using the “glm” function in the R package “stats”. These analytes (metabolites and lipoproteins) were used as the predictors (*x*), while the dichotomic variable indicating the status (i.e., CTR or dn^2^PD) was used as the dependent variable (*y*) to be predicted.

The fitted values obtained for each analyte and for each subject were used to estimate areas under the ROC curves (AUC values, using the “colAUC” function of the R package “caTools”). Subsequently, the fitted regression models built on the training set were used to predict probabilities of samples in the validation cohort (values between 0 and 1) to be classified as CTR or dn^2^PD (“predict.glm” function in the R package “stats”). These predicted probabilities were used to calculate AUC values for the validation cohort that were further compared with AUC values reported for the training cohort.

### Reporting summary

Further information on research design is available in the Nature Research Reporting Summary linked to this article.

## Supplementary information

Supplementary Information

Supplementary Data 1

Reporting Summary

## Data Availability

The metabolite and lipoprotein concentrations dataset used and/or analyzed during the current study are available as Supplementary Data Table.

## Code availability

Statistical codes used will be shared by request from any qualified investigator.
