# Supplementary material for: Metabolite and lipoprotein profiles reveal sex-related oxidative stress imbalance in de novo drug-naive Parkinson’s disease patients
Source: NPJ Parkinsons Dis. 2022 Feb 8;8:14. doi: 10.1038/s41531-021-00274-8 (PMC8826921; doi:10.1038/s41531-021-00274-8)

Supplementary Information

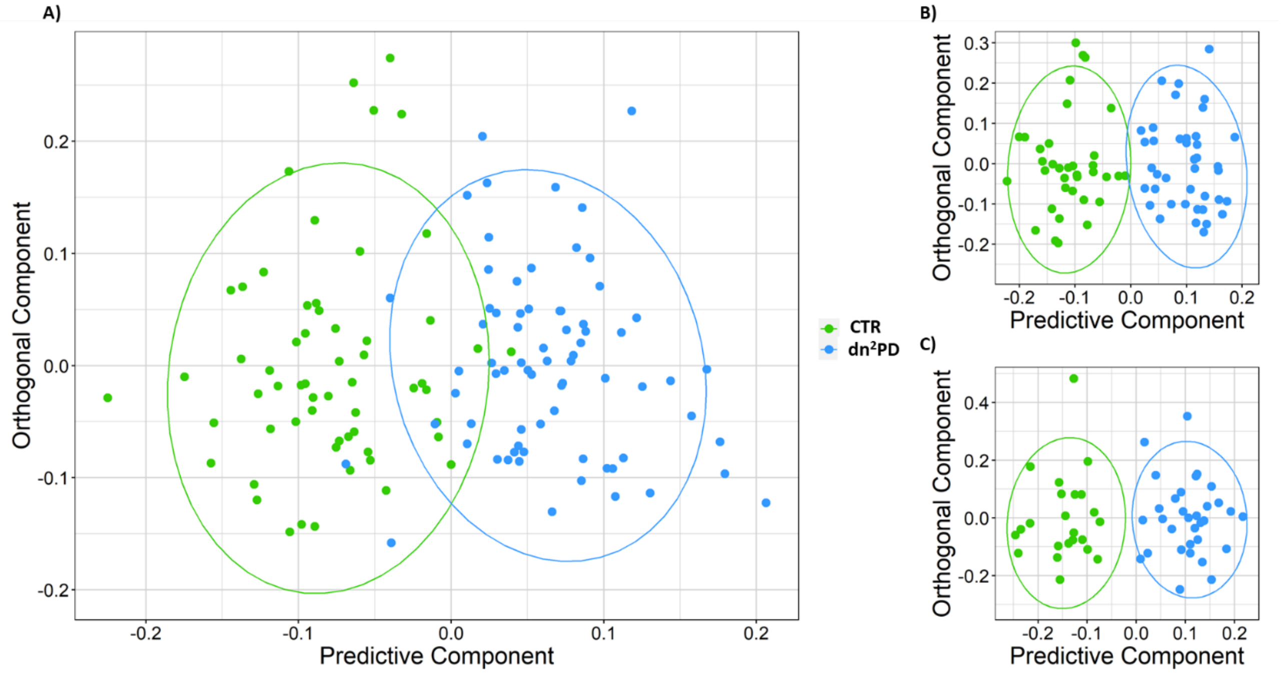

Supplementary Figure 1. OPLS-DA score plots of bucketed 1D-NOESY ^1H NMR serum spectra. **A**) OPLS-DA score plot of overall training cohort; **B**) OPLS-DA score plot of male training subjects; **C**) OPLS-DA score plot of female training subjects. Colours coded for individual status: CTR (green dots) and dn^{2}PD patients (blue dots). Ellipses indicate the confidence interval (*P*-value < 0.05) for each group.

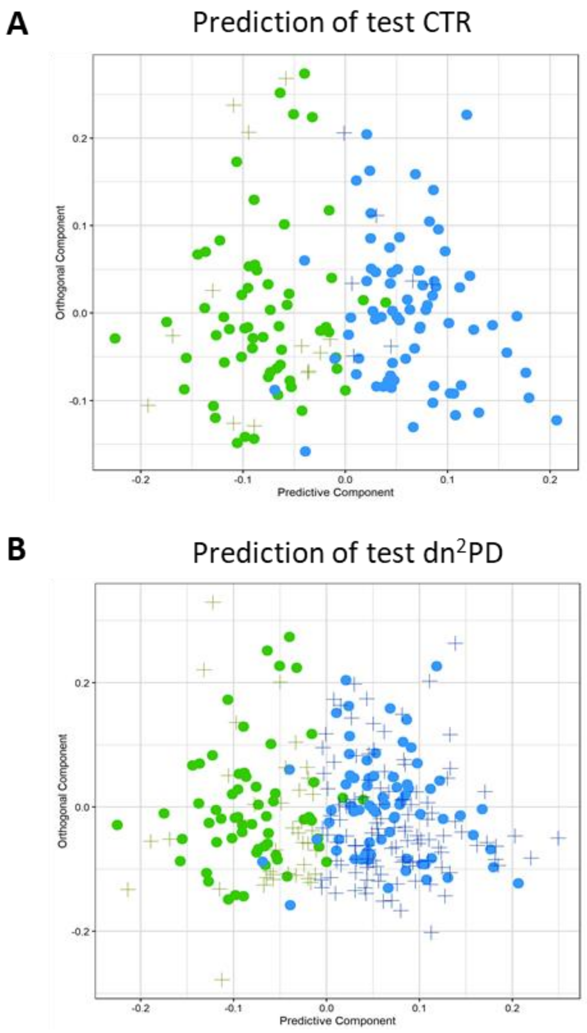

Supplementary Figure 2. OPLS-DA score plot of training model discriminating CTR (green dots) from dn^{2}PD patients (blue dots). “+” light green symbol: validation samples of true healthy controls; “+” dark blue symbol: validation samples of true dn^{2}PD.

Supplementary Table 1. Complete Log_2(Fold-Change) ratios of univariate groups' comparison. Positive Log_2FC values refer to higher metabolite/lipoprotein levels in dn^2PD patients compared to CTR group of training samples. *P*-value, FDR and Effect size are also reported. Quantified metabolites and lipoproteins are coloured in green and orange respectively.

	MALES				FEMALES			
	p-value	*FDR*	*Effect size*	*$Log_2(FC)$*	*p-value*	*FDR*	*Effect size*	*$Log_2(FC)$*
Acetic-acid	$3.76 \cdot 10^{1}$	0.6639	negligible	-0.1312	0.3093	0.5981	small	-0.1829
Acetoacetic-acid	$4.10 \cdot 10^{1}$	0.6917	negligible	0.0704	0.5959	0.8229	negligible	0
Acetone	$5.79 \cdot 10\text{-}0^{6}$	0.0008	large	0.9092	0.3977	0.6784	negligible	0.4854
Alanine	$5.22 \cdot 10^{1}$	0.7653	negligible	-0.0329	0.0222	0.3221	medium	0.182
Citric-acid	$4.57 \cdot 10^{2}$	0.1466	small	0.192	0.9252	0.9252	negligible	-0.0273
Creatine	$4.32 \cdot 10^{1}$	0.7015	negligible	-0.415	0.8779	0.9092	negligible	0.1278
Creatinine	$7.35 \cdot 10^{1}$	0.8747	negligible	-0.0076	0.5331	0.773	negligible	-0.0284
Dimethylsulfone	$3.32 \cdot 10^{1}$	0.611	negligible	-0.0589	0.2113	0.5699	small	-0.1926
Formic-acid	$6.19 \cdot 10^{2}$	0.1885	small	0.1325	0.7581	0.8332	negligible	-0.0566
Glucose	$5.60 \cdot 10^{1}$	0.7853	negligible	-0.0634	0.7137	0.8294	negligible	-0.0144
Glutamine	$2.44 \cdot 10^{1}$	0.4982	small	0.024	0.5057	0.7718	negligible	-0.0148
Glycine	$1.37 \cdot 10^{2}$	0.0674	small	0.13	0.701	0.8294	negligible	-0.0024
Histidine	$4.61 \cdot 10^{2}$	0.1466	small	0.0982	0.2287	0.5699	small	0.0704
Isoleucine	$9.34 \cdot 10^{1}$	0.9461	negligible	0.0224	0.2526	0.5699	small	0
Lactic-acid	$2.12 \cdot 10^{2}$	0.0892	small	0.2499	0.3554	0.6441	small	0.2619
Leucine	$7.08 \cdot 10^{1}$	0.8728	negligible	-0.0599	0.1802	0.5699	small	0.1288
Methionine	$1.27 \cdot 10^{1}$	0.3289	small	0.4066	0.7001	0.8294	negligible	0.3576
N,N dimethylglycine	$1.09 \cdot 10^{1}$	0.2929	small	0	0.2751	0.5699	small	0.2224
Ornithine	$3.17 \cdot 10^{4}$	0.0054	large	1.1543	0.0859	0.4635	small	0.585
Phenylalanine	$2.73 \cdot 10^{5}$	0.0019	large	0.2338	0.0959	0.4635	small	0.1669
Pyruvic-acid	$4.76 \cdot 10^{1}$	0.7399	negligible	-0.0975	0.1803	0.5699	small	0.1844
Sarcosine	$9.08 \cdot 10^{1}$	0.9326	negligible	0	0.7758	0.8332	negligible	0.1699

Succinic-acid	$1.60 \cdot 10^{1}$	0.3818	small	0.415	0.715	0.8294	negligible	0
TrimethylamineNoxide	$3.38 \cdot 10^{1}$	0.6116	negligible	0.3692	0.4369	0.7038	negligible	-0.4639
Tyrosine	$7.00 \cdot 10^{1}$	0.8728	negligible	0.012	0.0142	0.3221	medium	0.1813
Valine	$2.89 \cdot 10^{1}$	0.5502	negligible	0.0455	0.0832	0.4635	small	0.1699
3-Hydroxybutyric-acid	$5.73 \cdot 10^{2}$	0.178	small	0.6234	0.2633	0.5699	small	0.6234
ApoA1	$5.45 \cdot 10^{1}$	0.7811	negligible	0.0197	0.6295	0.9805	negligible	0.0031
ApoA2	$5.24 \cdot 10^{1}$	0.7653	negligible	0.019	0.3524	0.9805	small	-0.1024
ApoB100	$7.49 \cdot 10^{4}$	0.0054	medium	-0.1879	0.6295	0.9805	negligible	-0.0497
Chol	$2.94 \cdot 10^{3}$	0.0175	medium	-0.1295	0.4426	0.9805	negligible	-0.0829
Tg	$4.41 \cdot 10^{1}$	0.7078	negligible	-0.1073	0.5706	0.9805	negligible	0.2642
ApoB100/ApoA1	$7.33 \cdot 10^{4}$	0.0054	medium	-0.2808	0.8981	0.9805	negligible	-0.0809
LDLChol/HDLChol	$4.46 \cdot 10^{4}$	0.0054	medium	-0.3539	0.798	0.9805	negligible	-0.0119
ApoA1-HDL	$4.59 \cdot 10^{1}$	0.728	negligible	0.0447	0.7329	0.9805	negligible	0.0045
ApoA1-HDL1	$3.94 \cdot 10^{1}$	0.6782	negligible	0.0519	0.7074	0.9805	negligible	0.0535
ApoA1-HDL2	$4.63 \cdot 10^{1}$	0.728	negligible	0.0645	0.4128	0.9805	negligible	-0.0195
ApoA1-HDL3	$1.95 \cdot 10^{1}$	0.4503	small	0.1467	0.6908	0.9805	negligible	-0.0461
ApoA1-HDL4	$4.91 \cdot 10^{1}$	0.7439	negligible	-0.0434	0.798	0.9805	negligible	-0.0115
ApoA2-HDL	$5.99 \cdot 10^{1}$	0.8321	negligible	0.0073	0.2824	0.9805	small	-0.0867
ApoA2-HDL1	$7.43 \cdot 10^{1}$	0.8747	negligible	-0.0697	0.3841	0.9805	negligible	0.0664
ApoA2-HDL2	$9.09 \cdot 10^{1}$	0.9326	negligible	-0.0021	0.2323	0.9805	small	-0.1723
ApoA2-HDL3	$7.43 \cdot 10^{1}$	0.8747	negligible	0.0051	0.2862	0.9805	small	-0.1025
ApoA2-HDL4	$6.55 \cdot 10^{1}$	0.8588	negligible	-0.0019	0.5278	0.9805	negligible	-0.0857
Chol-HDL	$4.29 \cdot 10^{1}$	0.7015	negligible	0.0315	0.8857	0.9805	negligible	0.0377
Chol-HDL1	$8.76 \cdot 10^{1}$	0.9279	negligible	-0.0534	0.8062	0.9805	negligible	0.0024
Chol-HDL2	$2.26 \cdot 10^{1}$	0.4715	small	0.1463	0.9117	0.9805	negligible	-0.081
Chol-HDL3	$1.29 \cdot 10^{1}$	0.3289	small	0.1679	0.7671	0.9805	negligible	0.0387
Chol-HDL4	$8.19 \cdot 10^{1}$	0.9078	negligible	-0.0198	0.4949	0.9805	negligible	-0.0482
FreeChol-HDL	$7.91 \cdot 10^{1}$	0.8904	negligible	-0.0406	0.899	0.9805	negligible	-0.0626
FreeChol-HDL1	$7.79 \cdot 10^{1}$	0.8839	negligible	-0.1228	0.8244	0.9805	negligible	-0.1274

FreeChol-HDL2	$2.27\cdot10^{1}$	0.4715	small	0.1155	0.7265	0.9805	negligible	-0.2852
FreeChol-HDL3	$8.27\cdot10^{1}$	0.9098	negligible	0.0316	0.3842	0.9805	negligible	-0.1567
FreeChol-HDL4	$9.42\cdot10^{1}$	0.9461	negligible	-0.0979	0.2785	0.9805	small	-0.0639
Pho-HDL	$2.14\cdot10^{1}$	0.4632	small	0.1126	0.7414	0.9805	negligible	-0.0317
Pho-HDL1	$6.74\cdot10^{1}$	0.8677	negligible	0.0187	0.7542	0.9805	negligible	0.138
Pho-HDL2	$1.60\cdot10^{1}$	0.3818	small	0.1447	0.8062	0.9805	negligible	0.015
Pho-HDL3	$1.27\cdot10^{1}$	0.3289	small	0.1285	0.4683	0.9805	negligible	-0.0846
Pho-HDL4	$9.46\cdot10^{1}$	0.9461	negligible	-0.0102	0.4014	0.9805	negligible	-0.0771
Tg-HDL	$6.14\cdot10^{1}$	0.8441	negligible	0.0316	0.9663	0.9933	negligible	0.0555
Tg-HDL1	$2.61\cdot10^{1}$	0.5188	small	0.309	0.9049	0.9805	negligible	-0.1337
Tg-HDL2	$5.09\cdot10^{1}$	0.7579	negligible	0.0652	0.932	0.993	negligible	0.1736
Tg-HDL3	$8.15\cdot10^{1}$	0.9078	negligible	0.0161	0.6389	0.9805	negligible	-0.1746
Tg-HDL4	$4.92\cdot10^{1}$	0.7439	negligible	-0.0037	0.8112	0.9805	negligible	0.0083
LDL	$1.60\cdot10^{4}$	0.0033	large	-0.2816	0.4014	0.9805	negligible	-0.0526
LDL1	$2.65\cdot10^{1}$	0.5193	small	0.0264	0.5143	0.9805	negligible	-0.1119
LDL2	$8.51\cdot10^{2}$	0.2367	small	-0.2226	0.5706	0.9805	negligible	-0.204
LDL3	$1.95\cdot10^{2}$	0.0892	small	-0.2771	0.6537	0.9805	negligible	-0.0447
LDL4	$6.63\cdot10^{4}$	0.0054	medium	-0.4911	0.9933	0.9933	negligible	0.0046
LDL5	$6.91\cdot10^{4}$	0.0054	medium	-0.3697	0.899	0.9805	negligible	-0.0437
LDL6	$2.37\cdot10^{2}$	0.0892	small	-0.1844	0.7801	0.9805	negligible	-0.025
ApoB-LDL	$1.60\cdot10^{4}$	0.0033	large	-0.2816	0.4014	0.9805	negligible	-0.0526
ApoB-LDL1	$2.61\cdot10^{1}$	0.5188	small	0.0261	0.5143	0.9805	negligible	-0.1123
ApoB-LDL2	$8.61\cdot10^{2}$	0.2367	small	-0.2227	0.5706	0.9805	negligible	-0.2038
ApoB-LDL3	$2.01\cdot10^{2}$	0.0892	small	-0.2769	0.6537	0.9805	negligible	-0.0445
ApoB-LDL4	$6.63\cdot10^{4}$	0.0054	medium	-0.4907	0.9864	0.9933	negligible	0.0047
ApoB-LDL5	$6.91\cdot10^{4}$	0.0054	medium	-0.3696	0.8914	0.9805	negligible	-0.0435
ApoB-LDL6	$2.37\cdot10^{2}$	0.0892	small	-0.1845	0.7801	0.9805	negligible	-0.0254
Chol-LDL	$1.22\cdot10^{4}$	0.0033	large	-0.2585	0.5447	0.9805	negligible	-0.0792
Chol-LDL1	$6.41\cdot10^{2}$	0.1908	small	-0.203	0.5034	0.9805	negligible	-0.0567

Chol-LDL2	$1.59 \cdot 10^{1}$	0.3818	small	-0.2222	0.5938	0.9805	negligible	-0.1301
Chol-LDL3	$3.65 \cdot 10^{2}$	0.1274	small	-0.2337	0.666	0.9805	negligible	-0.054
Chol-LDL4	$7.20 \cdot 10^{4}$	0.0054	medium	-0.4684	0.8511	0.9805	negligible	0.1122
Chol-LDL5	$6.57 \cdot 10^{4}$	0.0054	medium	-0.356	0.9933	0.9933	negligible	-0.0161
Chol-LDL6	$2.24 \cdot 10^{2}$	0.0892	small	-0.1809	0.966	0.9933	negligible	0.0751
FreeChol-LDL	$3.54 \cdot 10^{4}$	0.0054	medium	-0.2955	0.5938	0.9805	negligible	-0.0877
FreeChol-LDL1	$1.40 \cdot 10^{1}$	0.3502	small	-0.0601	0.2098	0.9805	small	-0.2078
FreeChol-LDL2	$6.32 \cdot 10^{1}$	0.845	negligible	0.0308	0.4176	0.9805	negligible	-0.1883
FreeChol-LDL3	$2.04 \cdot 10^{1}$	0.4632	small	-0.1153	0.701	0.9805	negligible	-0.1668
FreeChol-LDL4	$3.13 \cdot 10^{3}$	0.0179	medium	-0.3157	0.9728	0.9933	negligible	-0.0544
FreeChol-LDL5	$1.28 \cdot 10^{3}$	0.008	medium	-0.3072	0.7717	0.9805	negligible	-0.0463
FreeChol-LDL6	$3.94 \cdot 10^{2}$	0.1311	small	-0.1485	0.7718	0.9805	negligible	0.0207
Pho-LDL	$1.11 \cdot 10^{4}$	0.0033	large	-0.2493	0.4477	0.9805	negligible	-0.0766
Pho-LDL1	$2.12 \cdot 10^{1}$	0.4632	small	-0.0547	0.4407	0.9805	negligible	-0.1133
Pho-LDL2	$2.10 \cdot 10^{1}$	0.4632	small	-0.1665	0.5477	0.9805	negligible	-0.1395
Pho-LDL3	$3.34 \cdot 10^{2}$	0.1209	small	-0.2053	0.5909	0.9805	negligible	-0.0673
Pho-LDL4	$1.21 \cdot 10^{3}$	0.0079	medium	-0.4287	0.8578	0.9805	negligible	0.0742
Pho-LDL5	$5.42 \cdot 10^{4}$	0.0054	medium	-0.3376	0.9796	0.9933	negligible	-0.0449
Pho-LDL6	$3.38 \cdot 10^{2}$	0.1209	small	-0.148	0.8311	0.9805	negligible	0.0221
Tg-LDL	$5.84 \cdot 10^{3}$	0.0298	medium	-0.2006	0.1804	0.9805	small	-0.0796
Tg-LDL1	$7.24 \cdot 10^{1}$	0.8747	negligible	-0.1901	0.2824	0.9805	small	-0.0337
Tg-LDL2	$1.78 \cdot 10^{1}$	0.4169	small	-0.0723	0.314	0.9805	small	-0.0796
Tg-LDL3	$1.17 \cdot 10^{3}$	0.0079	medium	-0.337	0.4683	0.9805	negligible	-0.018
Tg-LDL4	$4.51 \cdot 10^{3}$	0.0248	medium	-0.3554	0.3613	0.9805	negligible	-0.1209
Tg-LDL5	$4.81 \cdot 10^{3}$	0.0255	medium	-0.3827	0.3524	0.9805	small	-0.0844
Tg-LDL6	$7.70 \cdot 10^{2}$	0.2201	small	-0.1525	0.7783	0.9805	negligible	-0.0953
IDL	$2.09 \cdot 10^{2}$	0.0892	small	-0.203	0.5734	0.9805	negligible	-0.1757
ApoB-IDL	$2.09 \cdot 10^{2}$	0.0892	small	-0.2026	0.5733	0.9805	negligible	-0.1751
Chol-IDL	$2.30 \cdot 10^{2}$	0.0892	small	-0.3398	0.8914	0.9805	negligible	-0.0026

FreeChol-IDL	$3.89 \cdot 10^{2}$	0.1311	small	-0.3172	0.8645	0.9805	negligible	0.0234
Pho-IDL	$7.70 \cdot 10^{2}$	0.2201	small	-0.2223	0.6207	0.9805	negligible	0.2168
Tg-IDL	$4.11 \cdot 10^{1}$	0.6917	negligible	-0.2446	0.6573	0.9805	negligible	0.4129
VLDL	$8.51 \cdot 10^{1}$	0.9155	negligible	0.1036	0.482	0.9805	negligible	0.1889
ApoB-VLDL	$8.47 \cdot 10^{1}$	0.9155	negligible	0.1032	0.4736	0.9805	negligible	0.1889
Chol-VLDL	$6.85 \cdot 10^{1}$	0.8728	negligible	0.0786	0.3919	0.9805	negligible	0.4222
Chol-VLDL1	$3.33 \cdot 10^{1}$	0.611	negligible	-0.167	0.3127	0.9805	small	0.8156
Chol-VLDL2	$5.53 \cdot 10^{1}$	0.7832	negligible	-0.0374	0.4895	0.9805	negligible	0.4758
Chol-VLDL3	$8.39 \cdot 10^{1}$	0.9155	negligible	0.1751	0.6389	0.9805	negligible	0.5032
Chol-VLDL4	$6.43 \cdot 10^{1}$	0.8519	negligible	0.1302	0.8325	0.9805	negligible	0.2311
Chol-VLDL5	$3.57 \cdot 10^{1}$	0.6385	negligible	0.1269	0.216	0.9805	small	0.2701
FreeChol-VLDL	$7.55 \cdot 10^{1}$	0.8747	negligible	0.1019	0.3554	0.9805	small	0.4975
FreeChol-VLDL1	$2.28 \cdot 10^{1}$	0.4715	small	-0.2898	0.4895	0.9805	negligible	0.6905
FreeChol-VLDL2	$7.47 \cdot 10^{1}$	0.8747	negligible	-0.0663	0.4225	0.9805	negligible	0.3991
FreeChol-VLDL3	$9.09 \cdot 10^{1}$	0.9326	negligible	0.1877	0.4225	0.9805	negligible	0.7304
FreeChol-VLDL4	$7.67 \cdot 10^{1}$	0.8773	negligible	0.1473	0.4948	0.9805	negligible	0.3756
FreeChol-VLDL5	$7.08 \cdot 10^{1}$	0.8728	negligible	0.4245	0.3862	0.9805	negligible	0.0589
Pho-VLDL	$6.32 \cdot 10^{1}$	0.845	negligible	0.0493	0.4611	0.9805	negligible	0.6681
Pho-VLDL1	$3.13 \cdot 10^{1}$	0.5888	negligible	-0.3037	0.556	0.9805	negligible	0.6083
Pho-VLDL2	$5.46 \cdot 10^{1}$	0.7811	negligible	-0.0674	0.4631	0.9805	negligible	0.3496
Pho-VLDL3	$8.64 \cdot 10^{1}$	0.9217	negligible	0.1694	0.5618	0.9805	negligible	0.3899
Pho-VLDL4	$9.13 \cdot 10^{1}$	0.9326	negligible	0.2092	0.6056	0.9805	negligible	0.1179
Pho-VLDL5	$7.35 \cdot 10^{1}$	0.8747	negligible	0.1541	0.2256	0.9805	small	0.2103
Tg-VLDL	$6.25 \cdot 10^{1}$	0.845	negligible	-0.0334	0.4407	0.9805	negligible	0.7592
Tg-VLDL1	$3.93 \cdot 10^{1}$	0.6782	negligible	-0.3019	0.4508	0.9805	negligible	0.6854
Tg-VLDL2	$7.08 \cdot 10^{1}$	0.8728	negligible	0.0204	0.2811	0.9805	small	0.4298
Tg-VLDL3	$8.84 \cdot 10^{1}$	0.9297	negligible	0.2029	0.4426	0.9805	negligible	0.3832
Tg-VLDL4	$6.62 \cdot 10^{1}$	0.8608	negligible	0.1536	0.5821	0.9805	negligible	0.243
Tg-VLDL5	$2.82 \cdot 10^{1}$	0.5441	negligible	0.1664	0.3181	0.9805	small	0.2834

Supplementary Table 2. Performances of the OPLS-DA models built to discriminate dn²PD male patients (n=123) from CTR male subjects (n=44). CV OPLS-DA model built on the matrix of metabolites and lipoproteins and the model built on the matrix of bucketed spectra, to discriminate cases from CTRs, are compared in the table. Sensitivity, specificity and accuracy, calculated according to the standard definition, are reported.

performances	Metabolites and lipoproteins			Bucketed spectra		
	Mean %	Min %	Max %	Mean %	Min %	Max %
**Sensitivity**	77.2	68.8	84.3	74.9	59.1	86.4
**Specificity**	61.4	45.5	75.6	79.4	70.4	88.6
**Accuracy**	73.05	65.6	79.62	77.2	65.9	86.4

Supplementary Figure 3. Importance of variables ranked by VIP (Variable Importance in Projection score) calculated from the OPLS-DA model built on quantified metabolites and lipoproteins (performance reported in Supplementary Table 2).

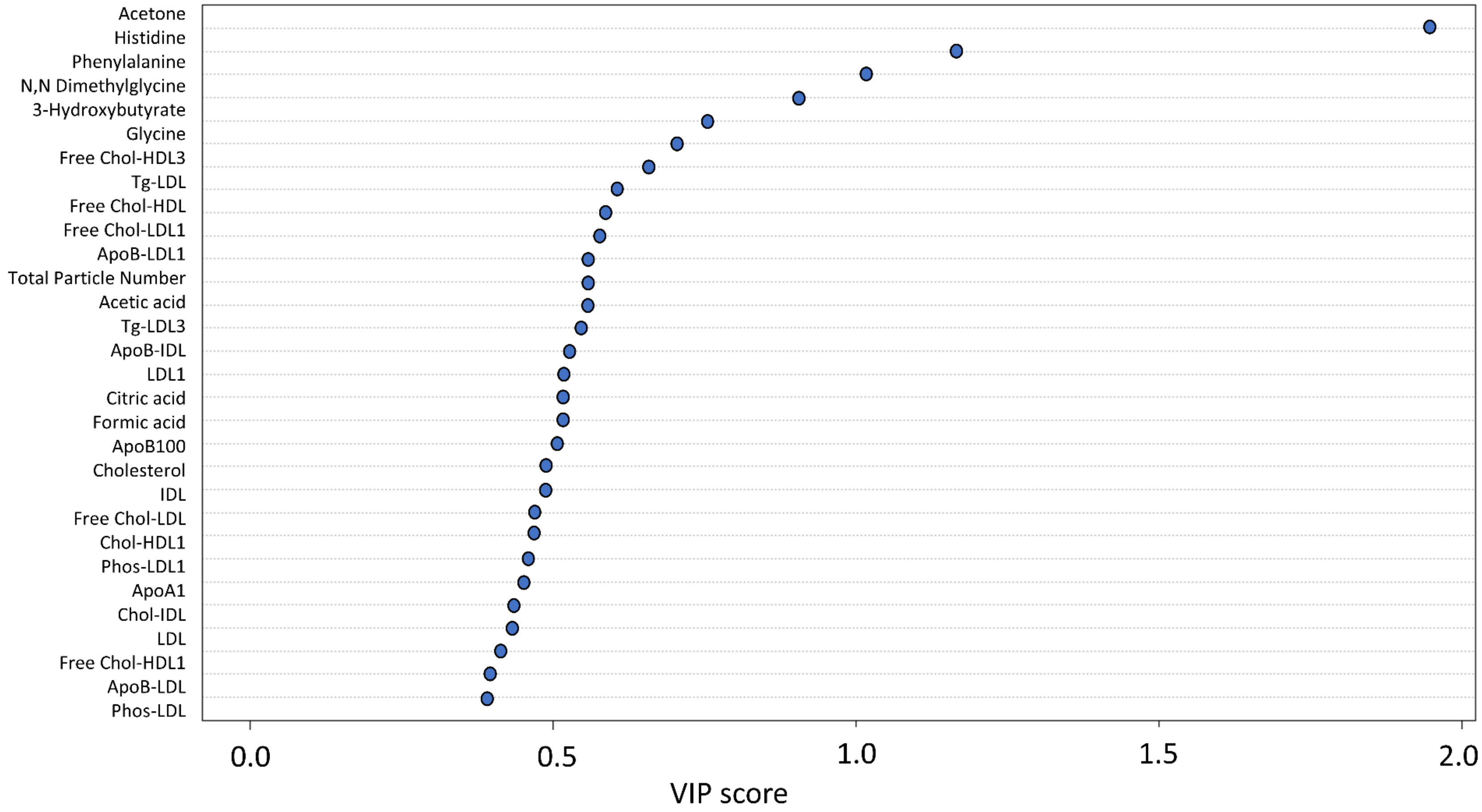

Supplement: Supplementary file 1 — Supplementary Information [file 41531_2021_274_MOESM1_ESM.pdf]
