# Supplementary material for: Metabolite and lipoprotein profiles reveal sex-related oxidative stress imbalance in de novo drug-naive Parkinson’s disease patients
Source: NPJ Parkinsons Dis. 2022 Feb 8;8:14. doi: 10.1038/s41531-021-00274-8 (PMC8826921; doi:10.1038/s41531-021-00274-8)
Supplement: Supplementary file 3 — Reporting Summary [file 41531_2021_274_MOESM3_ESM.pdf]

nature portfolio

Corresponding author(s): Luchinat Claudio and Mollenhauer Brit

Last updated by author(s): 09/12/2021

Reporting Summary

Nature Portfolio wishes to improve the reproducibility of the work that we publish. This form provides structure for consistency and transparency in reporting. For further information on Nature Portfolio policies, see our Editorial Policies and the Editorial Policy Checklist.

Statistics

For all statistical analyses, confirm that the following items are present in the figure legend, table legend, main text, or Methods section.

n/a	Confirmed	
☐	☒	The exact sample size (*n*) for each experimental group/condition, given as a discrete number and unit of measurement
☐	☒	A statement on whether measurements were taken from distinct samples or whether the same sample was measured repeatedly
☐	☒	The statistical test(s) used AND whether they are one- or two-sided<br>*Only common tests should be described solely by name; describe more complex techniques in the Methods section.*
☐	☒	A description of all covariates tested
☐	☒	A description of any assumptions or corrections, such as tests of normality and adjustment for multiple comparisons
☐	☒	A full description of the statistical parameters including central tendency (e.g. means) or other basic estimates (e.g. regression coefficient) AND variation (e.g. standard deviation) or associated estimates of uncertainty (e.g. confidence intervals)
☐	☒	For null hypothesis testing, the test statistic (e.g. *F*, *t*, *r*) with confidence intervals, effect sizes, degrees of freedom and *P* value noted<br>*Give P values as exact values whenever suitable.*
☐	☒	For Bayesian analysis, information on the choice of priors and Markov chain Monte Carlo settings
☐	☒	For hierarchical and complex designs, identification of the appropriate level for tests and full reporting of outcomes
☐	☒	Estimates of effect sizes (e.g. Cohen's *d*, Pearson's *r*), indicating how they were calculated

Our web collection on statistics for biologists contains articles on many of the points above.

Software and code

Policy information about availability of computer code

Data collection	NMR Topspin 3.2 software (Bruker BioSpin), AssureNMR (version 2.2) software (Bruker BioSpin), AVANCE Bruker IVDr (Clinical Screening and In Vitro Diagnostics research, Bruker BioSpin) software
Data analysis	R (version 3.6.1) software

For manuscripts utilizing custom algorithms or software that are central to the research but not yet described in published literature, software must be made available to editors and reviewers. We strongly encourage code deposition in a community repository (e.g. GitHub). See the Nature Portfolio guidelines for submitting code & software for further information.

Data

Policy information about availability of data

All manuscripts must include a data availability statement. This statement should provide the following information, where applicable:

- Accession codes, unique identifiers, or web links for publicly available datasets
- A description of any restrictions on data availability
- For clinical datasets or third party data, please ensure that the statement adheres to our policy

The metabolite and lipoprotein concentrations dataset used and/or analysed during the current study are available as Supplementary Data Table.

Field-specific reporting

Please select the one below that is the best fit for your research. If you are not sure, read the appropriate sections before making your selection.

☒ Life sciences ☐ Behavioural & social sciences ☐ Ecological, evolutionary & environmental sciences

For a reference copy of the document with all sections, see nature.com/documents/nr-reporting-summary-flat.pdf

Life sciences study design

All studies must disclose on these points even when the disclosure is negative.

Sample size	Post-hoc power calculation was performed after sample collection.<br>t test power calculation on training cohort<br><br>dn2PD = 72<br>CTR = 59<br>Effect size = 0.4956538<br>sig.level = 0.05<br>power = 0.8<br>alternative = two.sided
Data exclusions	No data have been excluded
Replication	A validation cohort with the same characteristics of training cohort have been collected to verify and validate results. However non all the observation made in the training cohort can be reproduced with the validation cohort
Randomization	not relevant to our study
Blinding	not relevant to our study becase it is not a clinical trial

Reporting for specific materials, systems and methods

We require information from authors about some types of materials, experimental systems and methods used in many studies. Here, indicate whether each material, system or method listed is relevant to your study. If you are not sure if a list item applies to your research, read the appropriate section before selecting a response.

Materials & experimental systems

n/a	Involved in the study	
☒	☐	Antibodies
☒	☐	Eukaryotic cell lines
☒	☐	Palaeontology and archaeology
☒	☐	Animals and other organisms
☐	☒	Human research participants
☒	☐	Clinical data
☒	☐	Dual use research of concern

Methods

n/a	Involved in the study	
☒	☐	ChIP-seq
☒	☐	Flow cytometry
☒	☐	MRI-based neuroimaging

Human research participants

Policy information about studies involving human research participants

Population characteristics	The study population consists of a total of 329 German subjects, including dn2PD patients, advPD patients, and healthy CTR. In detail, patient cohorts were: 1) a training cohort, consisting of 72 dn2PD(age =65.1, male =40) and 59 CTR (age =64, male =64.5), for a total of 131 subjects from the baseline visit of the Kassel cohort; 2) an independent validation cohort consisting of samples from 156 dn2PD (age= 65, male =83), 20 CTR (age=71.7, male =8) and 22 advPD (age = 68.9, male=15) patients, for a total of 198 subjects, as part of the cross-sectional Kassel cohort.
Recruitment	Patients enrolled in this study were clinically phenotyped before sample collection. Phenotyping included 1.5 Tesla magnetic resonance imaging (MRI) to determine structural abnormalities, quantitative levodopa testing, smell identification test (Sniffin' sticks, Burghardt Messtechnik GmbH, Wedel, Germany), Mini Mental Status Examination (MMSE) followed by further cognitive testing and video-supported polysomnography to determine REM sleep behaviour disorder in a subset of patients. The phenotyping was done based on these results and in accordance to established criteria for PD (UK Brain Bank Criteria), Multiple System Atrophy (MSA), Dementia with Lewy bodies (DLB), Progressive Supranuclear Palsy (PSP), Corticobasal Degeneration (CBD), Alzheimer's disease and Frontotemporal Dementia (FTD). Subjects with marked vascular lesions in MRI indicative of a vascular comorbidity and subjects with normal pressure hydrocephalus by MRI were excluded.

Ethics oversight	The study was conducted according to the Declaration of Helsinki and with informed written consent provided by all subjects. The study was approved by the ethics committee of the Physician’s Board Hesse, Germany (Approval No. FF89/2008 for DeNoPa) and the University Medical Center Goettingen, Germany (Approval No. 9/7/04 and 36/7/02 for Kassel cohort).

Note that full information on the approval of the study protocol must also be provided in the manuscript.
